# Efficacy and tolerability of an ectoine mouth and throat spray compared with those of saline lozenges in the treatment of acute pharyngitis and/or laryngitis: a prospective, controlled, observational clinical trial

**DOI:** 10.1007/s00405-016-4060-z

**Published:** 2016-04-28

**Authors:** Dörte Müller, Torben Lindemann, Kija Shah-Hosseini, Olaf Scherner, Markus Knop, Andreas Bilstein, Ralph Mösges

**Affiliations:** 1 Institute for Medical Statistics, Informatics and Epidemiology (IMSIE), Faculty of Medicine, University of Cologne, Lindenburger Allee 42, 50931 Cologne, Germany; 2 bitop AG, Witten, Germany

**Keywords:** Ectoine mouth and throat spray, Saline lozenges, Acute laryngitis, Acute pharyngitis, Oral treatment, Pharyngeal treatment

## Abstract

The aim of this observational trial was to evaluate the efficacy and tolerability of a mouth and throat spray containing ectoine in the treatment of acute pharyngitis and/or laryngitis. The outcome was compared with control treatment using saline lozenges. This study was designed as a prospective, controlled, non-randomized, observational multicenter clinical trial and was conducted in Germany. The study population consisted of 95 patients. The decision for treatment with either spray or lozenges was based on the patients’ preference for pharyngeal or oral application. Investigators assessed symptoms specific to acute pharyngitis/laryngitis and determined the pharyngitis symptom score. Both patients and investigators evaluated the tolerability and efficacy of the treatment applied. Treatment with the spray showed higher efficacy, 1.95 ± 0.81 versus 1.68 ± 0.67 (investigators) and 1.97 ± 0.88 versus 1.57 ± 0.69 (patients, *p* < 0.05). Treatment with the spray resulted in significantly greater reduction of cervical lymph node swelling (*p* < 0.05), ∆ spray = 0.44 ± 0.62, ∆ lozenges = 0.21 ± 0.62. The lozenges showed some advantage in relieving cough, ∆ lozenges = 0.62 ± 0.94 versus ∆ spray = 0.44 ± 0.85. Both patients and investigators rated the tolerability of both medical devices as “good” to “very good”. Adverse events of mild to moderate severity were either possibly related or not related to the medical devices used. No serious adverse events occurred. Taken together, while the tolerability was consistent in both treatment groups, the ectoine-based spray showed superior efficacy in treating acute pharyngitis and/or laryngitis.

**Electronic supplementary material:**

The online version of this article (doi:10.1007/s00405-016-4060-z) contains supplementary material, which is available to authorized users.

## Introduction

Pharyngitis or sore throat is a frequently occurring condition that is mostly accompanied by infections of the respiratory system, such as tonsillitis, rhinopharyngitis, and tonsillopharyngitis. It is a typical example of diagnosed conditions whose signs and symptoms are shared by a variety of other disorders. They are, therefore, often combined under the term “pharyngitis”. The most common symptoms are fever, throat pain, headache, and “dry mouth”, often accompanied by swallowing difficulties. Milder and moderate forms of pharyngitis are treated symptomatically with analgesics, disinfection solutions, or lozenges containing anesthetics. If a bacterial infection cannot be ruled out, antibiotic treatment is indicated [1], which may decrease the duration of symptoms [2].

According to a German study from 1989, pharyngitis is responsible for nearly 2 % of patient consultations in general medical practices [3]. A study carried out over a time period of 10 years highlighted that a sore throat is diagnosed in 1.1 % of all consultations in a general medical practice [4]. During the timespan of the study, the investigators observed an increasing prevalence of pharyngitis, making it the eighth most common reason for seeking medical advice, with its incidence continuing to increase. However, patients seek medical attention only if they suffer from severe pain or, for example, because their illness also has an impact on their families [5, 6]. It is assumed that the estimated number of pharyngitis cases is much higher, because most patients contact neither a general practitioner nor an ENT specialist. These patients mainly engage in self-care with over-the-counter (OTC) medication.

Recently, the inflammation-reducing properties of ectoine have been demonstrated in several preclinical [7–9] and clinical studies involving different indications [10–13]. Ectoine increases both the stability and fluidity of biomembrane layers as demonstrated in biophysical experiments [14–16], leading to the increased stabilization of the epithelial barrier, the subsequent reduction in inflammation, and protection against stress [17]. The stabilization effect on the barrier function of the epithelia tissue has led to the hypothesis that ectoine increases the resistance of the pharyngeal mucosa and improves its recovery. During the last decade, ectoine has been used as the treatment for allergic rhinitis, rhinoconjunctivitis, and other diseases [10–13, 18].

A previous study, investigating the use of ectoine in patients suffering from acute rhinosinusitis yielded positive results [11]. The aim of this observational trial was, therefore, to evaluate the efficacy and tolerability of Ectoin^®^ spray for pharyngeal conditions in comparison to Emser^®^ Pastillen as active control.

On the OTC market, saline lozenges are intended to treat acute throat pain, sore throat, cough, and strain of voice. One kind of such lozenges contains Ems salt. This ingredient is said to facilitate healing and to promote cell function in general (product information for Emser Pastillen^®^ without menthol, http://www.emser.de).

## Methods

### Study design

This trial was a prospective, controlled observational study that enrolled a total of 95 patients between March and June 2014. Patients were not randomized to a treatment group; instead, the patients’ preference for spray or lozenges determined their treatment group. All patients enrolled in the seven ENT trial sites were aged 10 years or older. Patients were eligible if acute pharyngitis and/or laryngitis had persisted for one or two days prior to enrollment. The treatment observation period lasted a maximum of 10 days and included an initial visit (V1), an intermediate visit (V2), and a final visit (V3) after one week.

The present study was conducted according to the principles of Good Clinical Practice. Since the spray and the lozenges are available without prescription, approval by an ethics committee was not required. Nevertheless, the responsible ethics committee was consulted with respect to professional regulations.

### Medication

Treatment was administered under consideration of the instructions for use (IFU). Ectoine [(*S*)-2-methyl-1,4,5,6-tetrahydropyrimidine-4-carboxylic acid (CAS 96702-03-3)] is a low-molecular mass osmolyte and belongs to the group of extremolytes. According to the IFU for Ectoin^®^ Mouth and Throat Spray 1 % (bitop AG, Witten, Germany), one to two puffs of the spray are to be applied into the throat several times a day. Emser^®^ Pastillen consist solely of many types of ions. According to the IFU for Emser Pastillen^®^ (Siemens & Co., Bad Ems, Germany), one to two lozenges should be taken as required up to six times a day.

### Clinical assessment

At (V1), the patients’ general medical histories were recorded, which included clinically relevant concomitant diseases and medication as well as allergic reactions.

At all visits, the patients’ conditions were assessed with regard to hoarseness and swallowing difficulties. Investigators also determined the pharyngitis symptom score, which consisted of the symptoms swollen palatine tonsils, swollen cervical lymph nodes, fever, and cough. Single scores were as follows: none = 0, mild = 1, moderate = 2, severe = 3.

Furthermore, patients were asked to fill out a diary on a daily basis for a minimum of seven days to document symptom severity. General health conditions and pain due to a sore throat were documented on a visual analog scale, and the pharyngitis symptom score was determined. The use of the medical devices, concomitant, and rescue medication (paracetamol) was also documented.

Efficacy was evaluated both by investigators and by patients at V2 and V3 on a 4-point scale (score 3 = very good; score 2 = good; score 1 = satisfactory; score 0 = poor).

Tolerability was evaluated in analogy to the efficacy score (see above) at V2 and V3 by the investigators and patients. To evaluate sensory impressions and possible irritations, the spray was also evaluated at V1 using a sensory scale. The questionnaire was adapted based on the nasal spray sensory scale [19].

### Safety

To assure the safety of the medical devices, patients were asked at each visit to report any adverse events (AEs). The occurrence of all AEs was documented. Additionally, patients with contraindications listed in the IFU were excluded from this study. If a bacterial infection was suspected, the investigator prescribed an antibiotic treatment.

### Statistical analysis

The study was analyzed using SPSS 22 statistics software by SPSS Inc. Data were entered twice into the data base to reduce data entry errors, and checks for plausibility were performed. Unavailable data were treated as missing values or, for analysis of the primary endpoints, substituted by the last-value-carried-forward method. The primary endpoints were compared between treatment groups using the nonparametric Wilcoxon test. The level of significance was set to *α* = 5 % for all statistical tests. Demographic, anamnestic, and diagnostic data were evaluated in a descriptive way. These data were expressed in terms of frequency, mean value, standard deviation, median, minimum, and maximum for each of the two treatment groups.

The Wilcoxon test was used to detect significant differences between the baseline sum scores and the final sum scores during and after the treatment.

## Results

### Study population

In total, 95 patients participated in this study: 64 patients applied the spray and 31 patients took the lozenges. The spray group consisted of 46 female and 18 male patients. The lozenge group consisted of 17 female and 12 male patients; the sex of two patients was not documented (Table 1).

**Table 1:** Demographic data

	Spray	Lozenges
Number of patients	64	31
Sex (female/male)	46/18	17/12
n.d.	–	2
Mean age (years)	50.53 ± 18.39	47.1 ± 19.87

Patients who applied the spray were 50.3 ± 18.39 years old on average; patients who took the lozenges were an average of 47.1 ± 19.87 years. Overall, the patients’ ages ranged from 10 to 90 years.

In the spray group, investigators diagnosed acute pharyngitis in 42 patients, acute laryngitis in six patients, and both conditions in 11 patients. The diagnosis of acute pharyngitis and/or acute laryngitis was not documented for five patients in the spray group. In the group taking the lozenges, acute pharyngitis was diagnosed in 14 patients, acute laryngitis in six patients, and both conditions in 11 patients.

Furthermore, diseases typically accompanying pharyngitis and/or acute laryngitis such as tonsillitis, rhinitis, esophagopharyngeal reflux, bronchitis, and flu were recorded for all patients.

Seven out of 95 patients dropped out of the study (7.4 %). Since the study applied the last-value-carried-forward method for missing data, however, the data sets of these patients were still able to be included in the analysis. Reasons for early discontinuation varied (see Supplementary Table 1).

### General health

The investigator evaluated the general health of the patient at each visit using a 4-point scale from 0 (=very poor health condition) to 3 (=good health condition). The health of patients treated with the spray showed an earlier improvement than the control group. In the spray group, the mean improvement increased from 0.23 ± 0.57 at V2 to 0.35 ± 0.65 at V3. The control group showed no improvement at V2 (mean = 0.0 ± 0.76) or at V3 (0.25 ± 0.70). The general health of all patients improved to an identical mean score of 2.71 in both groups at V3, corresponding to a nearly “good health condition”. Compared with V1, patients treated with the spray improved by 14.8 %, patients treated with the lozenges improved by 10.1 %.

The patients’ diary ratings of their general health condition showed that patients treated with the spray improved significantly from Day 3 of treatment (*p* < 0.01). On Day 3, their mean improvement of 0.76 ± 1.90 was higher than the mean improvement of 0.08 ± 1.28 for the patients treated with the lozenges. On Day 7, the spray group showed a higher mean improvement of 1.60 ± 2.13 than that of the lozenge group (0.68 ± 1.91). The improvement in the lozenge group was significant after six days of treatment (*p* < 0.05). However, no significant differences between the two treatment groups could be observed.

The spray showed a numerical advantage with respect to swallowing difficulties throughout the treatment of seven days and also when assessed by investigators: the average improvements for the spray group of 0.75 ± 0.95 (V2) and 1.07 ± 1.02 (V3) were higher than the improvements of 0.48 ± 0.83 (V2) and 0.93 ± 0.55 (V3) for the lozenge group.

Sore throat symptoms were relieved similarly under both treatments. Patients evaluated the severity of their throat pain using a continuous scale ranging from 0 to 10, with 0 meaning no pain and 10 meaning the severest pain imaginable. Throughout a treatment course of seven days, the spray showed a numerical advantage in alleviating symptoms. On Day 3, the spray group showed a mean improvement of 1.01 ± 1.71, and on Day 7 it increased to 1.92 ± 2.37. In comparison, patients treated with lozenges had lower mean improvements of 0.82 ± 1.45 on Day 3 and 1.89 ± 1.81 on Day 7.

### Pharyngitis symptom score

At the beginning of the study, patients in both groups had mild symptoms as rated by the investigators. The severity of the symptoms at V1 in the groups did not differ significantly from each other. Both treatment groups showed very similar improvements between V1 and V2. Also, the improvements between V1 and V3 were nearly the same: 54.9 % in the lozenge group and 54.8 % in the spray group. Overall, the symptoms resolved over the course of the visits from mild at V1 to very mild or nearly no symptoms at V3 (Table 2 and Supplementary Table 3) with no significant difference between the groups.

**Table 2:** Pharyngitis symptom score evaluated by investigators—individual and summarized parameters

	Pharyngitis symptom score summarized	Swollen palatine tonsils	Swollen cervical lymph nodes	Fever	Cough
Spray					
Improvement V1 V2	0.70 ± 1.51	0.30 ± 0.69	0.34* ± 0.66	0.09 ± 0.54	−0.03 ± 0.84
Improvement V1 V3	1.43 ± 1.60	0.44 ± 0.69	0.44 ± 0.62	0.14 ± 0.52	0.44 ± 0.85
Lozenges					
Improvement V1 V2	0.69 ± 1.73	0.28 ± 0.75	0.03^ ± 0.62	0.13 ± 0.43	0.23 ± 0.86
Improvement V1 V3	1.50 ± 1.73	0.46 ± 0.51	0.21 ± 0.62	0.21 ± 0.68	0.62 ± 0.94

* *p* < 0.01

^^^ *p* < 0.05

The pharyngitis symptom score improvement rates derived from the diary also showed no significant difference between the two treatment groups. The group treated with the spray first exhibited significant improvement starting from Day 4 (*p* < 0.01) of the treatment, showing a mean improvement of 0.80 ± 1.94 versus 0.44 ± 2.10 for the lozenge group. The patients treated with lozenges showed significant improvement from Day 6 (*p* < 0.05) with a mean of 0.89 ± 1.97. On Day 7, the spray group improved by 51.4 % and the lozenge group by 50.7 %.

The individual parameters of the pharyngitis symptom score were as follows:

The investigators’ rating of “swollen palatine tonsils” yielded similar results for the spray and the lozenges (Table 2). The observed reduction in the swelling of palatine tonsils derived from the patients’ assessments showed a similar course as well (data not shown).

Patients treated with the spray had a significantly greater improvement of “swollen cervical lymph nodes” from V1 to V2 as reported by the investigators (*p* < 0.05; Table 2 and Supplementary Table 2). The average improvements in the spray group were 0.34 ± 0.66 at V2 and 0.44 ± 0.62 at V3. In contrast, treatment with lozenges resulted in lower mean improvements of 0.03 ± 0.62 at V2 and 0.21 ± 0.62 at V3.

The evaluation of the patients’ assessments within the treatment groups showed a significant reduction in cervical lymph node swelling after Day 5 of treatment with the spray (*p < 0.01) and after Day 6 of treatment with the lozenges (^*p* < 0.05). After seven days, symptoms improved more greatly under the treatment with spray (56.2 %) than under the lozenge treatment (33.3 %) (data not shown). There was no significant difference between the groups.

The symptom of fever improved similarly between V1 and V2 as well as between V2 and V3 in both groups. No significant changes were detected during the treatment (Table 2). This was true for both the investigators’ and the patients’ evaluations.

The lozenges had a numerical advantage in alleviating cough throughout the treatment (Table 2). The same trend was observed for the patients’ evaluations. On Day 7, cough improved by an average of 0.34 ± 1.01 in patients treated with the lozenges, whereas the patients using the spray showed a lower average improvement of 0.02 ± 1.04. However, none of the results differed significantly from each other.

### Efficacy rating by investigators and patients

**Fig. 1:**
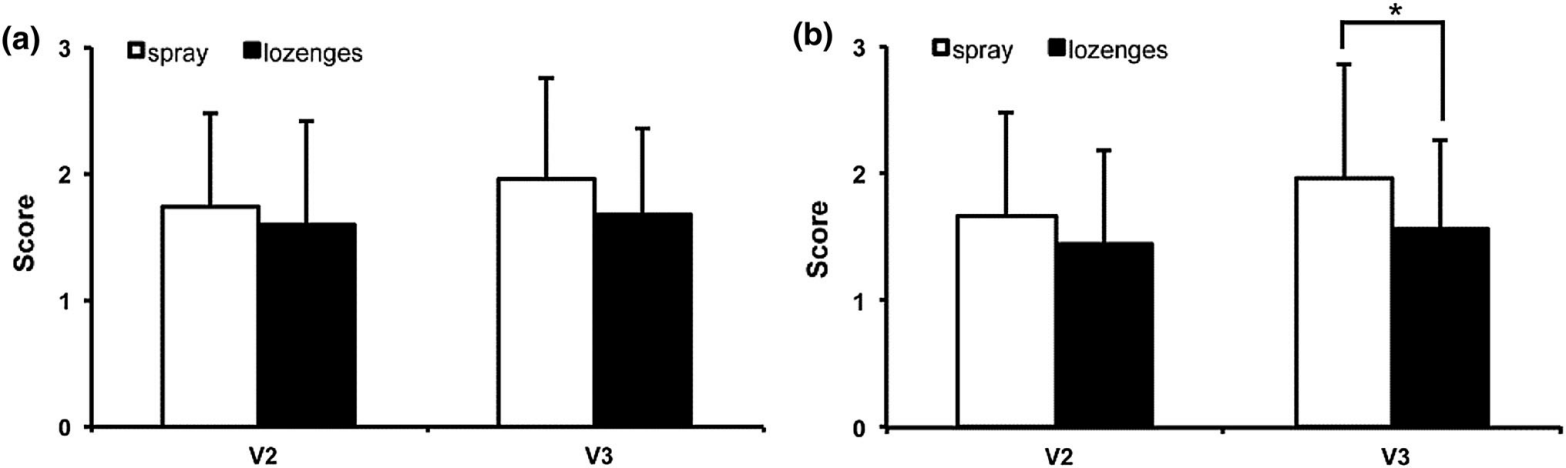
Efficacy of investigational products. Scores: 0 = poor, 1 = satisfactory, 2 = good, 3 = very good. * = *p* < 0.05. **a** Efficacy evaluated by investigators. **b** Efficacy evaluated by patients

The efficacy of the spray had a mean of 1.75 ± 0.73 (V2) and 1.95 ± 0.81 (V3) when evaluated by investigators and 1.66 ± 0.82 (V2) and 1.97 ± 0.88 (V3) when evaluated by patients (Fig. 1) which corresponds to an assessment of “good”. The evaluation of the lozenges by investigators resulted in mean values of 1.59 ± 0.83 (V2) and 1.68 ± 0.67 (V3), and the evaluation by patients resulted in 1.45 ± 0.74 (V2) and 1.57 ± 0.69 (V3). This corresponds to an assessment between “satisfactory” and “good”. At V3, the patients’ assessment of the spray was significantly different from that of the lozenges (*p* < 0.05, Fig. 1b).

### Tolerability and sensation of tasting flavor of the spray

Both the investigators and the patients rated the tolerability of the spray and the lozenges as “good” to “very good”.

The patients’ general sensory impression of the spray resulted in 76 % satisfaction. Patients were also satisfied with regard to irritation in general (90.6 % satisfaction) or tussive irritation (87.3 % satisfaction) immediately after applying the spray. Patients were pleased with the smell and taste of the spray, as demonstrated by their satisfaction rates of 73.1 to 87.7 %. The result of the entire questionnaire demonstrated 81.6 % overall satisfaction.

### Concomitant medication

In the spray group, 17.2 % (*n* = 11) of patients took concomitant medication at least once from Day 1 to Day 7, which was similar to the control group rate of 16.1 % (*n* = 5). The frequency of antibiotic intake was similar between the two groups: 10.9 % patients of the spray group and 12.9 % patients in the lozenge group took antibiotics.

### Rescue medication

On the first day of treatment, more patients in the spray group (24 %) took rescue medication (paracetamol) than in the lozenge group (15 %). In the middle of the treatment period on Day 4, the consumption of rescue medication decreased by 10 %, with 14 % patients in the spray group taking rescue medication. In the control group, the consumption of rescue medication on Day 4 was decreased by 5 %.

### Adverse events

In total, six mild to moderate adverse events were reported. In the spray group five AEs (7.8 %) occurred, whereof three were unlikely to be related, one AE was unrelated, and for one AE the relationship to the treatment medication could not be evaluated. In the control group, one patient (3.2 %) suffered from an AE, which was unlikely to be related. No serious adverse event (SAE) was reported.

## Discussion

This study compared two medical devices in the treatment of patients suffering from acute pharyngitis and/or laryngitis. The topical formulation Ectoin^®^ Mouth and Throat Spray 1 % was compared to oral treatment with Emser Pastillen^®^.

This study was designed as a prospective, controlled, nonrandomized observational study. Patients were allocated to the study groups based on their preferred treatment form; therefore, this study generates valuable data on daily medical practice. The data of the two treatment groups are well-balanced with respect to demographic data, history, symptoms, and health status prior to treatment. The study’s design and scope are comparable to those of other studies dealing with sore throat [20, 21].

The dropout rate up to the last visit was 6.3 % for the spray group and 9.7 % for the lozenge group. No dropout was due to a directly related AE. Altogether there were four possibly related AEs, three of which occurred in the spray group, and comprised headache, worsening of cough and sore throat, and nausea. In the control group, one patient suffered from nausea and subsequently dropped out. Since the patient population using the spray was twice as large as that taking the lozenges, the dropout rates for both medications are comparable. Taken together, the dropouts do not provide any indications for a noticeable problem with either medical device applied in this study. The frequency of dropouts is comparable to that reported in other studies dealing with sore throat [20, 21] or is considerably lower [22]. Patients participating in this clinical trial generally suffered from mild to moderate symptoms and had slightly reduced general health conditions. However, it can be assumed that the participants suffered severely from pharyngitis [5, 6].

Antibiotic use can be considered equal in the two groups: 10.9 % patients using the spray and 12.9 % patients taking the lozenges took antibiotics. The success of antibiotics in terms of the relief of symptoms is modest because antibiotics act only after three to four days and reduce the disease duration by half a day. For the most part, the prescription of antibiotics is unnecessary [2, 23, 24].

Since acute pharyngitis is mostly a self-limiting disease [1], it was necessary to prove the benefit of the novel ectoine-based spray. The considerably stronger amelioration of health conditions resulting from the treatment with the investigational product compared to the control product was confirmed by both the patients’ diary data and the physical examinations.

The clearly positive effect of the spray may underscore its suitability as a therapeutic alternative to antibiotics, because fewer and fewer people desire antibiotics according to previous studies [24]. The improvement of symptoms under the spray treatment also becomes evident in the patients’ assessments. The patients rated the efficacy of the spray (1.97 ± 0.88) significantly better than the efficacy of the lozenges (1.57 ± 0.69) (*p* < 0.05). The comparison of the need for rescue medication showed that 24 % of all patients in the investigational group and 15 % in the control group used rescue medication on the first day of the study. Because the values remained very similar in both groups after one day, they did not distort the final results. The higher value on Day 1 could be explained by slightly worse general health conditions in patients treated with the spray, i.e., 2.36 ± 0.57 versus 2.48 ± 0.51 for the lozenges.

The tolerability of both study medications was rated very similarly by both investigators and patients. The evaluations ranged mainly between “good” and “very good”, in which the spray displayed a numerical advantage compared to the lozenges.

## Conclusion

The treatment of acute laryngitis and/or pharyngitis symptoms with the ectoine-based spray demonstrates superiority to saline lozenges in improving the general health condition of patients. Moreover, the efficacy and tolerability profile of the spray compares very favorably with that of the lozenges. In addition, the ectoine-based spray has a positive safety profile and may therefore be considered a viable alternative for treating acute laryngitis and pharyngitis symptoms.

## Electronic supplementary material

Below is the link to the electronic supplementary material.

Supplementary material 1 (DOCX 28 kb)
