# Supplementary material for: Efficacy and tolerability of an ectoine mouth and throat spray compared with those of saline lozenges in the treatment of acute pharyngitis and/or laryngitis: a prospective, controlled, observational clinical trial
Source: Eur Arch Otorhinolaryngol. 2016 Apr 28;273:2591–7. doi: 10.1007/s00405-016-4060-z (PMC4974281; doi:10.1007/s00405-016-4060-z)
Supplement: Supplementary file 1 — Supplementary material 1 (DOCX 28 kb) [file 405_2016_4060_MOESM1_ESM.docx]

**Efficacy and tolerability of an ectoine mouth and throat spray compared with those of saline lozenges in treatment of acute pharyngitis and/or laryngitis: a prospective, controlled, observational clinical trial**

Dörte Müller^1^, Torben Lindemann^1^, Kija Shah-Hosseini^1^, Olaf Scherner^2^, Markus Knop^2^, Andreas Bilstein^2^, Ralph Mösges^1*^

^1^Institute for Medical Statistics, Informatics and Epidemiology (IMSIE), Faculty of Medicine, University of Cologne, Cologne, Germany

^2^bitop AG, Witten, Germany

*corresponding author (ralph@moesges.de)

**Supplementary Data**

**Table 1 . Reasons for dropping out**

| **Treatment group** | **N (%)** | **Reasons for dropping out** |
| --- | --- | --- |
| Ectoine based spray | 4 (6.3%) | - Nausea (V2) - Deterioration of patien's general condition (V2) - Improvement of patient’s general condition, lack of time, personal reasons (V3) - Dental treatment, infection of tooth (V3) |
| Saline lozenges | 3 (9.7%) | - Lack of time, personal reasons (V2) - Nausea (V2) - Job-related reasons (V3) |

**Table 2 . Swollen cervical lymph nodes**

| **Treatment  group** |  |  | **V1** | **V2** | **V3** | **Improvement V1 V2** | **Improvement V1 V3** |
| --- | --- | --- | --- | --- | --- | --- | --- |
| Ectoine based spray | N | Valid | 63 | 60 | 60 | 59 | 59 |
|  |  | Missing | 1 | 4 | 4 | 5 | 5 |
|  | **Mean** |  | **0.76** | **0.42** | **0.27** | **0.34*** | **0.44** |
|  | SD |  | 0.665 | 0.671 | 0.516 | 0.659 | 0.623 |
|  | Min. |  | 0 | 0 | 0 | -1 | -1 |
|  | Max. |  | 2 | 3 | 2 | 2 | 2 |
| Saline lozenges | N | Valid | 31 | 30 | 29 | 30 | 29 |
|  |  | Missing | 0 | 1 | 2 | 1 | 2 |
|  | **Mean** |  | **0.68** | **0.67** | **0.45** | **0.03*** | **0.21** |
|  | SD |  | 0.748 | 0.758 | 0.632 | 0.615 | 0.620 |
|  | Min. |  | 0 | 0 | 0 | -2 | -1 |
|  | Max. |  | 3 | 3 | 2 | 1 | 1 |

**Table 3. Pharyngitis symptom score**

| **Treatment group** |  |  | **V1** | **V2** | **V3** | **Improvement V1 V2** | **Improvement V1 V3** |
| --- | --- | --- | --- | --- | --- | --- | --- |
| Ectoine based spray | N | Valid | 59 | 55 | 58 | 53 | 54 |
|  |  | Missing | 5 | 9 | 6 | 11 | 10 |
|  | Mean |  | **2.61** | **1.85** | **1.09** | **0.70** | **1.43** |
|  | SD |  | 1.462 | 1.726 | 1.502 | 1.514 | 1.609 |
|  | Min. |  | 0 | 0 | 0 | -3 | -2 |
|  | Max. |  | 7 | 8 | 7 | 4 | 5 |
| Saline lozenges | N | Valid | 30 | 30 | 29 | 29 | 28 |
|  |  | Missing | 1 | 1 | 2 | 2 | 3 |
|  | Mean |  | **2.73** | **2.10** | **1.21** | **0.69** | **1.50** |
|  | SD |  | 1.617 | 1.709 | 1.373 | 1.734 | 1.732 |
|  | Min. |  | 0 | 0 | 0 | -5 | -3 |
|  | Max. |  | 7 | 7 | 6 | 3 | 5 |
